# Topological Insulator Films for Terahertz Photonics

**DOI:** 10.3390/nano12213779

**Published:** 2022-10-26

**Authors:** Kirill A. Kuznetsov, Sergey A. Tarasenko, Polina M. Kovaleva, Petr I. Kuznetsov, Denis V. Lavrukhin, Yury G. Goncharov, Alexander A. Ezhov, Dmitry S. Ponomarev, Galiya Kh. Kitaeva

**Affiliations:** 1 Faculty of Physics, Lomonosov Moscow State University, 119991 Moscow, Russia; 2 Ioffe Institute, 194021 Saint-Petersburg, Russia; 3 Kotelnikov IRE RAS (Fryazino Branch), 141190 Fryazino, Russia; 4 Institute of Ultra High Frequency Semiconductor Electronics of RAS, 117105 Moscow, Russia; 5 Prokhorov General Physics Institute of RAS, 119991 Moscow, Russia; 6 Moscow Institute of Physics and Technology, 141700 Dolgoprudny, Russia

**Keywords:** topological insulator, terahertz radiation, THz, third harmonic generation, photoconductive antenna, bismuth selenide, chalcogen-ordered tetradymite

## Abstract

We discuss experimental and theoretical studies of the generation of the third terahertz (THz) frequency harmonic in thin films of Bi_2_Se_3_ and Bi_2-x_Sb_x_Te_3-y_Se_y_ (BSTS) topological insulators (TIs) and the generation of THz radiation in photoconductive antennas based on the TI films. The experimental results, supported by the developed kinetic theory of third harmonic generation, show that the frequency conversion in TIs is highly efficient because of the linear energy spectrum of the surface carriers and fast energy dissipation. In particular, the dependence of the third harmonic field on the pump field remains cubic up to the pump fields of 100 kV/cm. The generation of THz radiation in TI-based antennas is obtained and described for the pump, with the energy of photons corresponding to the electron transitions to higher conduction bands. Our findings open up possibilities for advancing TI-based films into THz photonics as efficient THz wave generators and frequency converters.

## 1. Introduction

Over the past decade, the attention of many researchers around the world has been attracted to fundamentally new quantum materials—topological insulators (TIs)—which support stable (“topologically protected”) conducting surface states [1,2,3]. These states arise due to strong spin–orbit interaction, leading to the inversion of the band structure of the bulk material. In contrast to the Tamm states on the surfaces of “ordinary” (topologically trivial) materials, whose energy positions, conducting properties, and very existence depend on the surface potential, the emergence of surface states in TIs follows from the band structure topology and is not related to the surface morphology [2,3]. As a result, the surface states in TIs fill the whole band gap of the bulk material and remain metallic even in the presence of impurities and surface defects, as guaranteed by topological arguments.

An important feature of the topological surface carriers is that they are characterized by linear dispersion and behave as two-dimensional Weyl fermions (a subset of massless Dirac particles). The carriers are chiral, i.e., their spin projections are rigidly coupled to the momentum directions in the surface plane, which enables the efficient control of magnetization by charge current and vice versa and leads to the topological magnetoelectric effect in the THz spectral range [4]. Additionally, the surface carriers have a huge mobility of the order 10^5^ cm^2^/V·s [5]. 

In connection with the possible application of TIs in such areas as quantum information [6], spintronics [7], and THz photonics [8,9], the study of the properties of topological electronic states is of great importance [10]. In addition to using TIs as detectors of THz radiation [11,12,13,14], it could be very promising to apply them as base materials for efficient frequency converters of THz radiation [15,16] and full-fledged sources such as photoconductive antennas (PCAs) [17,18]. The generation of the third THz harmonic in Bi_2_Se_3_ TIs was first experimentally studied in Ref. [15]. The authors estimated the third harmonic generation efficiency as 1% for the incident field of 300 kV/cm and also observed electromagnetic transparency in the strong electric fields of the order of 1 MV/cm. A number of papers were devoted to the generation of THz radiation pulses due to the optical excitation of photocurrents in TI epitaxial films in the absence of an external bias electric field and the study of underlying mechanisms. In particular, Ref. [19] reported a circular anisotropy in the THz radiation distribution in Bi_2_Se_3_ and attributed it to the circular photon drag effect. Ref. [20] presented an experimental study of the mechanisms of transient THz emission from Bi_2_Te_3_ and concluded that the surface nonlinear currents dominate in the THz emission. Generation of THz radiation under the action of pulsed optical pumping has been recently investigated in TIs of double [21,22] and quaternary [23] chemical compositions. In Ref. [24], THz radiation was generated in Bi_2_Se_3_ and Cu-doped Bi_2_Se_3_ single crystals. The authors suggested that surface Dirac fermions are responsible for THz radiation due to the strong dependence of the radiation power on the carrier density.

Further development of topological-insulator-based THz photonics can involve increasing the efficiency of conversion to higher THz harmonics. The challenging task here is to choose or design TIs that demonstrate the highest nonlinear response but do not exhibit saturation effects in the required range of pump fields. Another topical direction is the use of TIs as active materials for PCAs [25]. Here, it is important to comprehensively study the generation in PCAs based on TIs with various pump sources, including such common ones as the Ti-Sa laser, to reveal the mechanisms of hot carrier relaxation and to numerically simulate generation processes in such sources. The paper is devoted to the consistent analysis of such devices based on TIs.

The paper is organized as follows. In Section 2, we develop a kinetic theory of the third harmonic generation by two-dimension carriers and present a description of the THz radiation generation in photoconductive antennas. Section 3 outlines the materials and experimental techniques. In Section 4, we present and discuss the experimental results on third harmonic generation and THz field generation. Section 5 summarizes the main results of the paper.

## 2. Theory

### 2.1. Kinetic Theory of THz Third Harmonic Generation

Here, we develop a kinetic theory of third harmonic generation by free carriers on the surfaces of TIs. The theory takes into account both the momentum and energy relaxation of the carriers, since the corresponding relaxation times are in the sub-ps range [16] and are comparable to the period of THz field. The current analysis extends previous considerations of the non-linear response of two-dimensional Dirac fermions done for graphene in collision-free [26,27] and one-relaxation-time [28,29] approximations. 

In the kinetic approach, electrons are described by the distribution function f(p,t), which satisfies the Boltzmann equation:

(1) $$\frac{\partial f}{\partial t}+eE(t)\cdot \nabla_{p}f=\mathrm{St}f$$

where e is the electron charge; E(t)=Eexp(−iωt)+c.c. is the electric field of the incident radiation; **E** and *ω* are the field amplitude and frequency, respectively; $\nabla_{p}=\partial /\partial p$; p is the momentum; and Stf is the collision integral that describes the electron gas relaxation. The approach of the kinetic equation (1) is valid provided the electron gas conductivity $\sigma \gg e^{2}/\hbar$ and the field frequency ω≪〈ε〉/ℏ, where ℏ is the reduced Planck constant and 〈ε〉 is the mean kinetic energy of electrons.

We solve the Boltzmann Equation (1) by expanding the field-induced correction δf(p,t) to the distribution function in the Fourier series in the time domain [30]:

(2) $$\delta f(p,t)=f^{\left(0\right)}+[f^{\left(1\right)}\exp(-i\omega t)+c.c.]+[f^{\left(2\right)}(-2i\omega t)+c.c.]+\ldots$$

where *c.c.* stands for complex conjugation. 

The correction to the distribution function of the first order in the electric field oscillates at ω-frequency and satisfies the equation

$$-i\omega f^{\left(1\right)}+eE(t)\cdot \nabla_{p}f^{\left(0\right)}=\mathrm{St}f^{\left(1\right)}$$

where $f^{\left(0\right)}$ is the equilibrium distribution function. It follows that the correction $f^{\left(1\right)}$ contains only the first angular harmonic in the momentum space. Accordingly, the collision integral $\mathrm{St}f^{\left(1\right)}$ in the relaxation time approximation assumes the form $-f^{\left(1\right)}/\tau_{1}$, where $\tau_{1}$ is the momentum relaxation time (relaxation time of the first angular harmonic of the distribution function). The straightforward solution of the above equation gives

(3) $$f^{\left(1\right)}=-e\tau_{1}(\omega )E\cdot \nabla_{p}f^{\left(0\right)}$$

where $\tau_{1}(\omega )=\tau_{1}/(1-i\omega \tau_{1})$. 

The second-order correction $f^{\left(2\right)}$ contains zero and second angular harmonics in the momentum space and is found from the equation

$$-2i\omega f^{\left(2\right)}+eE(t)\cdot \nabla_{p}f^{\left(1\right)}=\mathrm{St}f^{\left(2\right)}$$

Its solution in the relaxation time approximation has the following form:

(4) $$f^{\left(2\right)}=-e\tau_{e}(2\omega )E\cdot \bar{\nabla_{p}f^{\left(1\right)}}-e\tau_{2}(2\omega )E\cdot [\bar{\nabla_{p}f^{\left(1\right)}}-\nabla_{p}f^{\left(1\right)}]$$

where the top line denotes averaging over the directions of the momentum p, $\tau_{2}(2\omega )=\tau_{2}/(1-2i\omega \tau_{2})$, $\tau_{e}(2\omega )=\tau_{e}/(1-2i\omega \tau_{e})$, $\tau_{2}$ is the relaxation time of the second angular harmonic, and $\tau_{e}$ is the time of thermalization and energy relaxation.

The electric current at the triple frequency, which induces the outgoing third-harmonic radiation, is determined by $f^{\left(3\right)}$,

(5) $$j_{3\omega }=eg\sum_{p}vf^{\left(3\right)},$$

where v=∂ε/∂p is the electron velocity, ε is the energy, and g is the factor of possible surface, spin, or valley degeneracy (g=1 for a single surface of TI). The correction $f^{\left(3\right)}$ satisfies the equation

$$-3i\omega f^{\left(3\right)}+eE(t)\cdot \nabla_{p}f^{\left(2\right)}=\mathrm{St}f^{\left(3\right)}$$

and contains the first and third angular harmonics. The contribution to $f^{\left(3\right)}$, which leads to the electric current, is described by the first angular harmonic and, therefore, is contained in the term $-e\tau_{1}(3\omega )E\cdot \nabla_{p}f^{\left(2\right)}$. This yields the equation

(6) $$j_{3\omega }=-ge^{2}\sum_{p}v\tau_{1}(3\omega )(E\cdot \nabla_{p}f^{\left(2\right)}).$$

In particular, in the approximation of the relaxation times, which are independent of the energy, and for a linearly polarized radiation, Equation (6) gives

(7) $$j_{3\omega }=ge^{4}\tau_{1}(\omega )\tau_{1}(3\omega )EE^{2}\sum_{p}\frac{\partial^{2}\varepsilon }{\partial p_{x}^{2}}\left[\tau_{e}(2\omega )\bar{\frac{\partial^{2}f_{0}}{\partial p_{x}^{2}}}+\tau_{2}(2\omega )\left(\frac{\partial^{2}f_{0}}{\partial p_{x}^{2}}-\bar{\frac{\partial^{2}f_{0}}{\partial p_{x}^{2}}}\right)\right].$$

For parabolic energy spectrum, the sum over p in the equation above vanishes. This means that the third-harmonic generation in 2D systems with the parabolic spectrum occurs in the quasi-classical regime, provided the relaxation times depend on energy.

For linear energy spectrum $\varepsilon =v\;\left|p\right|$, similar calculations yield the non-vanishing current:

(8) $$j_{3\omega }=\frac{-ge^{4}v^{2}EE^{2}\tau_{1}(\omega )\tau_{1}(3\omega )}{8\pi \hbar^{2}E_{F}}\left[\tau_{e}(2\omega )+\frac{\tau_{2}(2\omega )}{2}\right],$$

where *E_F_* is the Fermi energy. 

In TIs based on bismuth and antimony chalcogenides such as Bi_2_Se_3_, the Dirac point is close to the valence band, and the Fermi energy of surface electrons can be quite large (of the order of the band gap). According to Equation (8), the efficiency of third harmonic generation, for a given electric field amplitude *E*, decreases in 2D Dirac systems with large Fermi energy. At the same time, the cubic dependence $j_{3\omega }\propto E^{3}$ holds as far as the relaxation times are not affected by electron gas heating (or, alternatively, the inequality $\omega \tau_{1}>>1$ is fulfilled) and the distribution of surface electrons remains degenerate. The latter also suggests that the peak energy gained by an electron at the cycle of the THz field, which is estimated as $\left|ev\tau_{1}(\omega )\right|E$, is less than the Fermi energy. As it has been recently measured, the relaxation of hot electrons in topological surface states is very fast [16]. The reason could be the interaction with optical phonons [31,32,33] or the efficient transfer of energy between surface and bulk carriers due to Coulomb interaction [34]. For fast energy relaxation, $\tau_{e}\sim \tau_{1}$, and efficient dissipation of heat from the surface region, the regime $j_{3\omega }\sim E^{3}$ can be optimistically extended up to the electric fields $E\sim E_{F}/\left|ev\tau_{1}(\omega )\right|$ giving rise the current amplitude $j\sim eE_{F}^{2}/\left(v\hbar^{2}\right)$. This demonstrates that 2D Dirac systems with large Fermi energy and fast energy relaxation can be highly efficient for frequency multiplication in the THz spectral range.

### 2.2. Generation of the THz Radiation in Photoconductive Antennas

Here, we use the simple model by Jepsen et al. [35,36], based on the Drude-Lorentz carrier transport theory. This theory well describes the generation of THz pulses emitted by small photoconductive semiconductor antennas. It is based on the system of differential Equation (9), given below. The system solutions represent theoretical time dependences of the electron concentration in the conduction band *n*(*t*), and the electron velocity *v*(*t*), obtained without taking into account the influence of the electrode parameters on the emitted THz field:

(9) $$\begin{array}{l} \frac{dn(t)}{dt}=-\frac{n(t)}{\tau_{c}}+\frac{\alpha }{hf_{p}}I(t)\\ \frac{dv(t)}{dt}=-\frac{v}{\tau_{1}}+\frac{e}{m^{*}}E_{m} \end{array}$$

where *E_m_* is an electric field at the location of charge carriers, and *I*(*t*) is the intensity of the laser source, determined by the expression:

(10) $$I(t)=I_{l}(1-R)\exp\left(-\frac{2t^{2}}{t_{p}^{2}}\right),$$

where *τ_c_* is the electron capture time, *τ*_1_ is the momentum relaxation time, *R* is the pump reflection coefficient, α is the absorption coefficient at the pump frequency, *f_p_* is the laser frequency, *t_p_* is the pump laser pulse duration, *h* is the Planck’s constant, *e* is the electron charge, and m^*^ is an electron effective mass. The electric field at the location of the charge carriers can be found from the expression:

(11) $$E_{m}=E_{b}-\frac{P(t)}{\eta \varepsilon },$$

where *P*(*t*) is the polarization, *E_b_* is the bias electric field, *η* is the screening coefficient, and *ε* is the dielectric function of the photoconductive material. At large screening factors *η*, the field *E_m_* becomes equal to *E_b_* so that

(12) $$E_{m}=E_{b}=\frac{U_{b}}{W},$$

where *U_b_* is the bias voltage, and *W* is the antenna’s gap. The relation of current density to the concentration *n*(*t*) and the velocity *v*(*t*) of charge carriers is described as j(t)=en(t)v(t). In turn, the time derivative of the current density determines the strength of the emitted THz field.

However, the resulting spectrum of the antenna is also greatly influenced by the geometry of the electrodes. It is known from experiments that, by varying parameters and the type (spiral, dipole, bow-tie, etc.) of the antenna electrodes, it is possible to radically change the THz generation spectrum [37]. Therefore, it is required to take into account these geometric factors. We have limited ourselves by studying a dipole-type antenna. This antenna can be considered as a resonator with a resonant frequency *f_0_* and a quality factor *Q*. The frequency transfer function of such a resonator is well described by the shape of a Lorentzian line with a width determined by the quality factor. Then, the resulting frequency dependence of the intensity spectrum of the THz field generated by the PCA *S*(*f*) can be found by the following empirical formula:

(13) $$S(f)\propto \frac{1}{{\left(f-f_{0}\right)}^{2}+{\left(\frac{f_{0}}{\sqrt{2}Q}\right)}^{2}}{\left(\int_{-\infty }^{\infty }\frac{\partial \left(n(t)v(t)\right)}{\partial t}\exp(-i2\pi ft)dt\right)}^{2},$$

where $f_{0}=c/2L\sqrt{(1+\tilde{\varepsilon })/2}$ is the resonance frequency of the dipole PCA [38], *L* is the length of the diagonal of the electrodes, $\tilde{\varepsilon }$ is an effective dielectric constant of the composite material of the thin film and substrate, and *Q* is the quality factor of the PCA, determined by its geometric dimensions.

## 3. Materials and Methods

### 3.1. Growth and Characterization

Rhombohedral films of binary (Bi_2_Se_3_) and quaternary (Bi_1.9_Sb_0.1_Te_2_Se) TIs were grown on 400-µm-thick (0001) sapphire substrates with a thin (5–20 nm) ZnTe buffer layer deposited in a horizontal quartz reactor at atmospheric pressure of hydrogen. It is known that in binary systems Bi(Sb)–Se(Te) there are different phases of the homologous series *m*Bi(Sb)_2·_*n*Bi_2_Se(Te)_3,_ where *m* and *n* are a number of Bi_2_, Sb_2_ and Bi_2_Se_3_ or Sb_2_Te_3_ blocks per unit cell. In a series of works by one of the authors of this work, the phase composition of films deposited at different temperatures was studied in detail in growth systems: trimethyl bismuth (BiMe_3_)—isopropyl selenide (iPro_2_Se)—hydrogen [39], trimethyl bismuth—diethyl telluride (Et_2_Te)—hydrogen [40] and trimethyl antimony (SbMe_3_)—diethyl telluride—hydrogen [41] on the (0001) sapphire substrates. It was shown by methods of X-ray diffraction and energy-dispersive X-ray spectroscopy that at temperatures higher than 440 °C, rhombohedral epitaxial films of the corresponding binary compounds are deposited with *m* = 0 и *n* = 1. Therefore, to ensure the growth of films of quaternary compounds of Bi_2−x_Sb_x_Te_3−y_Se_y_ stoichiometry, a temperature of 445 °C was used in this work.

Trimethyl bismuth BiMe_3_, SbMe_3_, ZnEt_2_, Et_2_Te, and iPro_2_Se were used as sources of bismuth, antimony, zinc, tellurium, and selenium, correspondingly. Stainless steel bubblers with organoelement compounds BiMe_3_, SbMe_3_, ZnEt_2_, Et_2_Te, and iPro_2_Se were thermostated at 0, −30, 10, 25, and 27 °C, respectively. The ZnTe buffer layers were grown in a single technological cycle with TI films at the same temperature of 445 °C. The total hydrogen flow was 1.0 L/min during the deposition of ZnTe buffer layers, and 0.5 L/min during the epitaxy of TI films. The ratio of elements of the V/VI group in the vapor phase was not lower than 10, and the total partial pressure of BiMe_3_+SbMe_3_ was kept close to 6 × 10^−5^ bar.

X-ray diffraction (XRD) was used to confirm the epitaxial nature of the grown films. To determine the elemental composition of the films, an X-MaxN energy-dispersive X-ray spectrometer (EDS), docked with an electron microscope, was used. The analysis was carried out utilizing the Oxford Instruments software. The following standards were used to standardize and optimize the line profiles of the characteristic radiation: Bi_2_Se_3_ (Bi-Mα and Se-Lα), Sb (Sb-Lα), ZnS (Zn-Lα), PbTe (Te-Lα), and Al_2_O_3_ (Al-Kα) crystals and O-Ka). Measurement of standards and analysis of samples were carried out under the same conditions at an accelerating voltage of 10 kV and an electron probe current of 1.4 nA. The spectrum accumulation time was set to 100 s. Under such conditions, the detection thresholds for all analyzed elements was 0.03–0.05 wt. %. As expected, in all BSTS films studied in this work, the ratio of elements of the V/VI groups was close to 2:3 and was within the limits of the detection thresholds. It should be emphasized that the composition of quaternary solid solutions cannot be determined from XRD spectra, and EDS analysis is required here.

The surface topography of the Bi_2_Se_3_ sample was obtained by atomic force microscopy (AFM) via a NT-MDT INTEGRA Prima scanning probe microscope (LLC “NT-MDT”, Moscow, Russia) operating in the semi-contact mode. Sample BSTS was studied by AFM AIST-NT Smart SPM with AIST-NT SPM Control software. Nanosensors PointProbePlus PPP-NCh-20, designed to operate in the semi-contact mode, were used. The radius of curvature of the tip end was 5–10 nm, and the oscillation amplitude of the free end of the cantilever far from the sample’s surface was chosen to be 15–20 nm. Before measurements, the samples were washed in isopropyl alcohol in an ultrasonic bath for 5 min and dried. In addition, for the samples without overhead electrodes, the side that was examined by AFM was studied by measuring the electrical conductivity. The resulting images were processed using the NT-MDT Image Analysis software. The results were used both to analyze the surface morphology and to obtain information about the surface roughness and film thickness. Note that our films were not thin enough for the surface Dirac states to completely disappear. As shown in Ref. [42], the Dirac cone of surface states disappears at 5 quantile thicknesses and less, while our TI samples are much thicker. Thus, topologically protected states exist on the film surface, and TI films can be treated as bulk samples.

**Figure 1:**
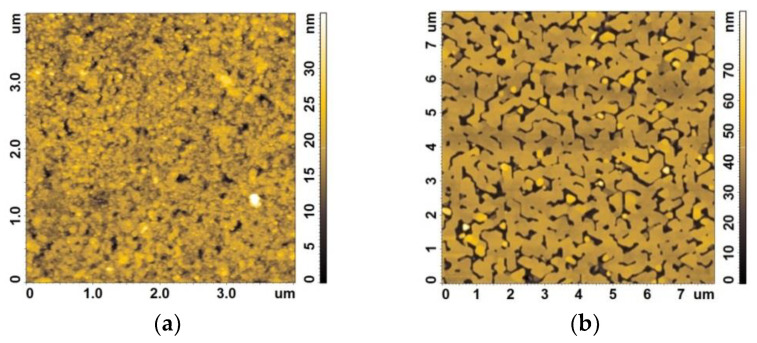
Atomic force microscopy images of Bi_2_Se_3_ (**a**) and BSTS (**b**) surfaces.

A typical AFM image of the Bi_2_Se_3_ film surface is shown in Figure 1a. From the analysis of the cross sections of the AFM images, it can be concluded that the “effective thickness” of the TI film is about 20 nm. In this case, the film itself is not completely continuous but contains a relatively small number of pores, the depth of which is equal to the film thickness. No crystallites with noticeable faceting were found on the surface; to characterize the quality of the surface, the roughness parameters were measured over an area of 16 μm^2^. The average roughness was 2.5 nm. The histogram of the distribution of surface heights in the same area was close to the normal distribution.

AFM studies of BSTS film on sapphire substrates showed that island film is formed at an early stage of growth by the Stranski–Krastanov mechanism (see Figure 1b). Hexagonal blocks are formed on the surface. The side faces of the blocks grow faster, and the completion of the merging of islands occurs at the height of about 40 nm. The surface roughness over the area of ~64 μm^2^ is 7.4 nm. The surface of the “islands” has a nm-size height terraces. The terraces have low contrast, because next to them there are the borders of the “islands”, the height of which is an order of magnitude greater. Further film deposition proceeds according to the two-dimensional (2D) growth regime. Upon transition to the 2D growth regime, only protrusions about 1 nm high are present on the surface, which are formed by five-layer Ch-Bi-Ch-Bi-Ch-Bi, where Ch is Te or Se. The surface roughness over the area of ~4 μm^2^ is 0.41 nm.

**Figure 2:**
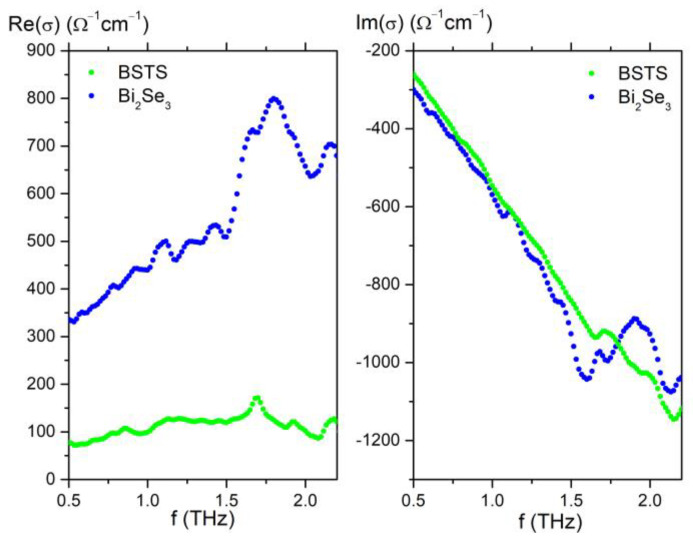
Frequency dependence of the real and imaginary parts of conductivity of Bi_2_Se_3_ and BSTS films.

To characterize the transmission spectrum of the samples, we used a time-domain spectrometer (TDS). An InGaAs THz emitter was used as a source of THz pulses for measurements. The transmitted terahertz pulses were measured by a ZnTe-based electro-optical sampling technique. It is known that the transmission function *T*(*f*) of a thin film with a thickness less than the radiation wavelength is directly related to the complex conductivity *G*(*f*) in accordance with the Tinkham’s formula [43,44]. The recalculated conductivity spectra are shown in Figure 2. The resonance near 1.6 THz occurs due to the interaction with the well-known dipole-active phonons. We have not considered ZnTe, because its conductivity is very low compared to that of the investigated TIs.

It can be seen that the conductivity is maximal in the Bi_2_Se_3_ sample. This fact is associated with a high concentration of bulk charge carriers due to the location of the Fermi level in the conduction band (Bi_2_Se_3_) [45]. On the contrary, in the BSTS sample, the bulk transfer is strongly suppressed, since the chemical composition is close to the Ren curve [45], and the Fermi level is located in the bulk bandgap. Although thicker films were studied in Ref. [45], our results for BSTS nano-films with compositions close to the Ren curve are in good agreement with the static resistivity data from Ref. [46].

### 3.2. Experimental Techniques

**Figure 3:**
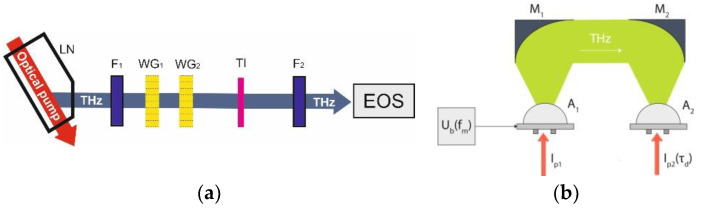
Sketches of experimental setups for studying the generation of the third terahertz harmonic (**a**) and the generation of terahertz radiation in the antenna (**b**).

To obtain a source of a strong THz field for frequency conversion, we used optical rectification of Ti-Sa laser pulses with tilted pulse front in a lithium niobate (LN) crystal. A band-pass filter F_1_ (Figure 3a) was installed after the crystal, which separated THz radiation at a fundamental frequency of 0.5 THz. The peak strength of the THz field was about of 100 kV/cm. To vary the THz pump field strength, a pair of wire grid polarizers (WG_1_ and WG_2_) was used; one of them was rotated on a motorized platform to control the transmitted power, and the other was fixed so that the polarization would be vertical. A band-pass filter F_2_ was installed after the TI sample, which suppressed THz radiation at the fundamental frequency and transmitted only its third harmonic at a frequency of 1.5 THz. The amplitude of the THz pump pulses and the third harmonic was measured electro-optically with a 2-mm-thick ZnTe crystal, using probing laser pulses of 100 fs duration (EOS).

To generate THz radiation, the PCA based on the TI BSTS was fabricated and studied. We used dipole antennas with annealed Ti/Pd/Au electrodes (50/150/200 nm thick) placed on the studied epitaxial films with a photoconductive gap *W* = 20 μm. To collimate the THz radiation, a hemispherical lens 0.5 cm in diameter made of high-resistance silicon (Tydex) was used as a lens coupler and attached to the TI-film. A schematic diagram of the TDS setup is shown in Figure 3b. A 100-femtosecond Ti-Sa laser with a wavelength of 0.78 μm was used as a laser pump source. The average pump power at the antenna-generator A_1_ in the operating mode was about 15 mW. The detection of THz radiation was performed by the antenna A_2_ Tera8-1 product (Menlo Systems); the signal from this was directed to the input of a lock-in amplifier. Parabolic mirrors M_1_ and M_2_ were used to collimate THz radiation. Instead of a typical beam chopper, we used the meander-shaped bias voltage modulation *U_b_* at a frequency of *f_m_* = 20 kHz.

## 4. Results and Discussion

### 4.1. Generation of the Third THz Harmonic

**Figure 4:**
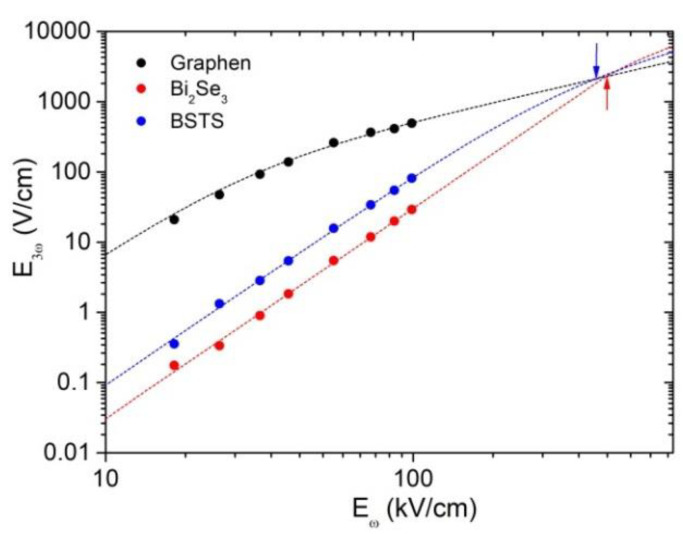
Third harmonic generation. Dependence of the peak amplitude of the outgoing third harmonic field on the pump field amplitude for graphene (black circles), BSTS (blue circles), and Bi_2_Se_3_ (red circles). Colored dashed lines: corresponding fittings. The points of intersection of the extrapolation curves are marked with arrows.

Figure 4 shows the dependencies of the peak power of the third harmonic generated in the Bi_2_Se_3_ and BSTS samples on the fundamental radiation field strength [16]. For comparison, we show also the results of measurements of *p*-doped graphene with a charge carrier concentration of about 10^13^ cm^−2^. Nowadays, graphene is the best material with the highest third harmonic conversion efficiency, reaching 1% and higher [47,48,49]. In our experiment, the highest field conversion efficiency in graphene is about 0.5%. The conversion efficiencies in the TIs Bi_2_Se_3_ and BSTS at 100 kV/cm are 0.03% and 0.08%, respectively. The amplitude of the third harmonic field in BSTS is about three times larger than that in Bi_2_Se_3_. The enhanced conversion efficiency in BSTS, where the Fermi energy of surface electrons is smaller, is in accordance with the theoretical result; see Equation (8) and the subsequent discussion. 

While the conversion efficiency achieved in Bi_2_Se_3_ and BSTS films is still lower than that in graphene, the TIs, in contrast to graphene, exhibits a purely cubic dependence of the third harmonic field on the pump field. This indicates that THz-induced nonlinear processes are far from saturation up to the pump fields of 100 kV/cm. We attribute these qualitatively different behaviors to a large difference in the relaxation times of carriers in graphene and TIs. In graphene, the energy dissipation is rather slow because of inefficient direct electron–phonon interaction and weak heat transfer from graphene to substrate [50,51]. In contrast, the energy relaxation of surface carriers in TIs occurs much faster, at the timescale of few hundred picoseconds, as was recently demonstrated for Bi_2_Te_3_ by means of THz pump-probe spectroscopy [16]. As follows from the theoretical considerations in Section 2.1, faster relaxation suggests larger range of the validity of the cubic law $E_{3\omega }\propto E_{\omega }^{3}$ and the possibility of reaching higher conversion efficiency. Therefore, one can expect that, at high values of the pumping field, the efficiency of conversion to the third harmonic of the TI will be comparable or even exceed that of graphene. 

To estimate the pump intensity, where the conversion efficiency of Bi_2_Se_3_ and BSTS would reach that of graphene, we extrapolated the pure cubic dependence $E_{3\omega }\propto E_{\omega }^{3}$ for Bi_2_Se_3_ and BSTS to higher fields. The validity of such an extrapolation is also supported by recent experiments by the Kovalev group [52], in which the cubic dependence is confirmed over the wide range of pump intensities. To approximate the saturating dependence of the third harmonic field strength in graphene, we use the empirical expected expression $E_{3\omega }\propto \frac{E_{\omega }^{3}}{1+E_{\omega }^{2}/E_{S}^{2}}$, where $E_{S}$ is the saturation strength of the cubic susceptibility. Extrapolating the theoretical dependences to the region of high pump field strengths $E_{\omega }>>100$ kV/cm, we estimate the intersection of these curves at the field strength $E_{\omega }^{c}=430$ kV/cm and $E_{\omega }^{c}=390$ kV/cm for Bi_2_Se_3_ and BSTS, respectively. Thus, we expect that TIs can be highly efficient frequency-converters of terahertz radiation in strong fields. By varying the chemical composition of TIs based on bismuth and antimony chalcogenides, one can tune the Fermi level and optimize the parameters of harmonic converters.

### 4.2. Photoconductive THz Antenna

**Figure 5:**
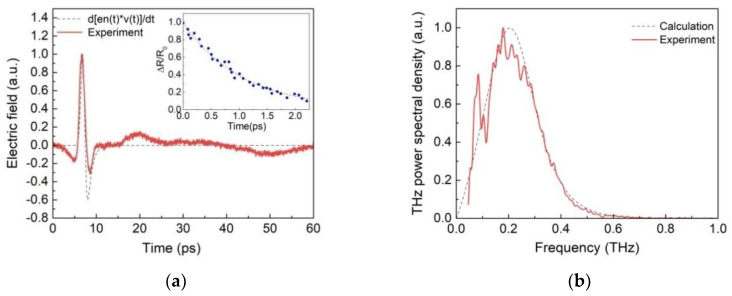
Waveform (**a**) and spectrum (**b**) of THz pulse from PCA. Inset: results of the pump-probe experiment.

The experimental data with the measured waveform are shown in Figure 5a. The corresponding frequency spectrum is shown in Figure 5b. The spectrum maximum is observed at 200 GHz; the linewidth at half maximum is also about 200 GHz. Expression (13) was used to approximate the experimental data. To find the temporal dependencies of the electron concentration *n*(*t*) and velocity *v*(*t*) of electrons, the system of Equation (9) was solved by the fourth-order Runge–Kutta numerical method using MATLAB software. The best approximation is achieved without taking into account the screening effect; therefore, the parameter η was considered equal to infinity. To determine the capture time *τ_c_*, the dynamics of excitation with energies above the bandgap were studied using the degenerate optical pump-optical probe spectroscopy (OPOP) scheme. The photon energy of optical pulses in OPOP was 1.5 eV, as in the experiment on THz generation in the photoconductive antenna. Results of the OPOP experiment are shown in the inset to Figure 5a, which shows the time dependence of the normalized induced change in the reflectance. Neglecting slower contributions, a single-exponential approximation yields the value for the capture time *τ_c_* = 0.93 ps. This is very close to the time scattering of photoexcited carriers into the surface states and lower bulk conduction band [53]. The following parameters were taken in further modeling of THz generation: *τ_c_* = 0.93 ps, *τ*_1_ = 0.1 ps, *τ_p_* = 0.1 ps, *R* = 0.31, *α* = 120,000 cm^−1^, *U_b_* = 15 V, *f_p_* = 3.8 × 10^14^ Hz, $\tilde{\varepsilon }$ = 14, and m* = 0.21 *m_e_* [54]. In order to find the quality factor of a dipole antenna with the parameters length *L* = 240 μm, gap *W* = 20 μm, and electrode width *d* = 40 μm, and according to Ref. [55], we find *Q* = 1.1. The calculation results are shown in Figure 5a,b by dashed lines. A fairly good agreement between the experimental data and predictions of the simplest THz generation model can be seen there.

Under optical pumping by Ti-Sa laser, the electrons passed through one C_1_ conduction band to another C_2_ [56] band, which is located higher in the energy diagram. The signal from the TI antenna turned out to be comparable in amplitude with the signal from an antenna thicker by an order of magnitude based on the InGaAs/InAlAs semiconductor heterostructure. This indicates that TI antennas are promising as commercial THz emitters, with laser pumping in the visible range.

## 5. Conclusions

We have discussed the results of the third THz harmonic generation in thin films of chalcogen-ordered tetradymites and the THz wave radiation in photoconductive antennas based on these topological insulators. The experiments are described by the developed kinetic theory of third harmonic generation by surface carriers and the Drude–Lorentz transport model of THz pulse generation applied to TIs.

The experiment shows that the dependence of the third harmonic field on the pump field in TIs is purely cubic up to the available fields of 100 kV/cm, with no evidence of saturation. Such a behavior is attributed to the fast energy relaxation of surface carriers. The experimental data and theoretical estimations allow us to speculate that the cubic dependence holds for stronger electric fields, and the efficiency of the frequency conversion in TIs can be even higher than that in graphene, where the highest efficiency has been achieved so far. The results on the generation of THz radiation pulses in photoconductive antennas show that thin films of TIs can be as efficient as conventional semiconductors with a much thicker active region.

We conclude that topologically non-trivial materials are promising candidates for applications in THz photonics.

## Data Availability

Not applicable.
